# *Lactobacillus reuteri* 1 Enhances Intestinal Epithelial Barrier Function and Alleviates the Inflammatory Response Induced by Enterotoxigenic *Escherichia coli K88 via* Suppressing the MLCK Signaling Pathway in IPEC-J2 Cells

**DOI:** 10.3389/fimmu.2022.897395

**Published:** 2022-07-14

**Authors:** Jingchun Gao, Shuting Cao, Hao Xiao, Shenglan Hu, Kang Yao, Kaiyong Huang, Zongyong Jiang, Li Wang

**Affiliations:** ^1^ State Key Laboratory of Livestock and Poultry Breeding, Ministry of Agriculture Key Laboratory of Animal Nutrition and Feed Science in South China, Guangdong Key Laboratory of Animal Breeding and Nutrition, Maoming Branch, Guangdong Laboratory for Lingnan Modern Agriculture, Institute of Animal Science, Guangdong Academy of Agricultural Sciences, Guangzhou, China; ^2^ College of Animal Sciences and Technology, Zhongkai University of Agriculture and Engineering, Guangzhou, China

**Keywords:** *Lactobacillus reuteri*, enterotoxigenic *Escherichia coli K88*, inflammatory response, intestinal epithelial barrier, MLCK signaling pathway

## Abstract

Intestinal epithelial barrier injury disrupts immune homeostasis and leads to many intestinal disorders. *Lactobacillus reuteri* (*L. reuteri*) strains can influence immune system development and intestinal function. However, the underlying mechanisms of *L. reuteri* LR1 that regulate inflammatory response and intestinal integrity are still unknown. The present study aimed to determine the effects of LR1 on the ETEC K88-induced intestinal epithelial injury on the inflammatory response, intestinal epithelial barrier function, and the MLCK signal pathway and its underlying mechanism. Here, we showed that the 1 × 10^9^ cfu/ml LR1 treatment for 4 h dramatically decreased interleukin-8 (IL-8) and IL-6 expression. Then, the data indicated that the 1 × 10^8^ cfu/ml ETEC K88 treatment for 4 h dramatically enhanced IL-8, IL-6, and tumor necrosis factor-α (TNF-α) expression. Furthermore, scanning electron microscope (SEM) data indicated that pretreatment with LR1 inhibited the ETEC K88 that adhered on IPEC-J2 and alleviated the scratch injury of IPEC J2 cells. Moreover, LR1 pretreatment significantly reversed the declined transepithelial electrical resistance (TER) and tight junction protein level, and enhanced the induction by ETEC K88 treatment. Additionally, LR1 pretreatment dramatically declined IL-8, IL-17A, IL-6, and TNF-α levels compared with the ETEC K88 group. Then, ETEC K88-treated IPEC-J2 cells had a higher level of myosin light-chain kinase (MLCK), higher MLC levels, and a lower Rho-associated kinase (ROCK) level than the control group, while LR1 pretreatment significantly declined the MLCK and MLC expression and enhanced ROCK level in the ETEC K88-challenged IPEC-J2 cells. Mechanistically, depletion of MLCK significantly declined MLC expression in IPEC-J2 challenged with *ETEC K88* compared to the si NC+ETEC K88 group. On the other hand, the TER of the si MLCK+ETEC K88 group was higher and the FD4 flux in the si MLCK+ETEC K88 group was lower compared with the si NC+ETEC K88 group. In addition, depletion of MLCK significantly enhanced Claudin-1 level and declined IL-8 and TNF-α levels in IPEC-J2 pretreated with LR1 followed by challenging with ETEC K88. In conclusion, our work indicated that *L. reuteri* LR1 can decline inflammatory response and improve intestinal epithelial barrier function through suppressing the MLCK signal pathway in the ETEC K88-challenged IPEC-J2.

## Introduction

Weaning stress is extremely serious during the initial post-weaning period, inducing alterations in intestinal morphology, disturbing intestinal microbiota, and inducing growth retardation, especially diarrhea brought by the invasion of pathogenic bacteria, particularly enterotoxigenic *Escherichia coli* (ETEC) (1–3). ETEC postweaning diarrhea, also known as postweaning enteric diarrhea, is a type of diarrhea that occurs during the weaned period (4, 5). Colibacillosis is a major cause of mortality and economic losses in global nursery pigs’ production (6). Thus, it is critical to identify a practical solution to alleviate the ETEC K88-induced intestinal disruption in weaned piglets.

*Lactobacillus reuteri* (*L. reuteri*) strains are critical parts of the commensal microbiota in the intestines of piglets that can influence the immune system development (7, 8). *L. reuteri* has been found to produce lactic acid, hydrogen peroxide (9, 10), and reutericyclin (11), which have antibacterial properties against some intestinal pathogens, such as *E. coli*. The intestinal immune system serves as an immunological barrier to improve mucosal barrier function (12). *L. reuteri* has been shown to regulate innate immune responses and intestinal barrier function (13, 14). A previous study reported that oral administration of *L. reuteri* I5007 enhanced T-cell differentiation and induced ileal cytokine production (15). Liu et al. found that *L. reuteri* DSM 17938 feeding of healthy newborn mice regulates immune responses while modulating gut microbiota and boosting beneficial metabolites (14). Previously, our group isolated *L. reuteri* LR1 from the feces of healthy weaned piglets, and its 16S rRNA sequence was submitted in the GenBank database (No. KT205306), and indicated that *L. reuteri* LR1 strain improved the intestinal epithelial barrier function (3, 16, 17). However, the underlying mechanisms of *L. reuteri* LR1 that regulate intestinal integrity and inflammatory response following challenge with ETEC K88 are still unknown. Myosin light chain kinase (MLCK) is a Ca^2+^ calmodulin-dependent serine/threonine kinase that constantly modulates actomyosin reorganization and cell contraction (18). Several studies reported that MLCK is involved in intestinal epithelial regulation, inflammation, and gastrointestinal disorders (18–20). It would be of great interest to focus on whether *L. reuteri* LR1 could prevent intestinal injury and inflammation induced by ETEC K88 *via* regulating the MLCK signaling pathway.

The present study aimed to determine the effects of LR1 on the ETEC K88-induced intestinal epithelial injury on the inflammatory response, intestinal epithelial barrier function, and the MLCK signal pathway and its underlying mechanism. The connection between the inflammatory response, intestinal epithelial barrier function, and the MLCK signal pathway provides insight into the development of nutritional strategies in the prevention of intestinal inflammation.

## Material and Methods

### Bacterial Strains and Growth Conditions

According to our previous reports (3, 16, 17), *L. reuteri* 1 (LR1) was isolated from the feces of a healthy 35-day-old weaned piglet and stored at our laboratory. The *L. reuteri* 1 strain was cultured overnight at 37°C in de Man Rogosa Sharpe (MRS) broth for 0 h, 4 h, 8 h, 12 h, 16 h, 20 h, and 24 h (Solarbio, Beijing, China). The ETEC K88 (ETEC) strain was cultivated in Luria-Bertani (LB) broth (Solarbio, Beijing, China) for 0 h, 4 h, 8 h, 12 h, 16 h, 20 h, and 24 h at 37°C with shaking at 170 rpm. The bacterial cultures were harvested by centrifugation for 5 min at 8,000 rpm and washed once with PBS. The bacteria were counted by dilution coating plate method and detected absorbance at OD_600nm_ at 0 h, 4 h, 8 h, 12 h, 16 h, 20 h, and 24 h (Bio-Tek, Winooski, USA).

### Cell Culture

IPEC-J2 was cultivated in DMEM/F-12 (Gibco) with 10% fetal bovine serum (Gibco) under a humidified atmosphere containing 5% CO_2_. Cells were tested negative for mycoplasma contamination before use. IPEC-J2 cells were treated with LR1 (1 × 10^9^ CFU/ml) for 4 h followed by treatment with ETEC K88 (1 × 10^8^ CFU/ml) groups for 4 h. To minimize the deviation, the control group and the other treatments had a consistent cell growth time. Briefly, as shown in **Supplementary Figure S1**, the IPEC-J2 cells (1×10^5^ cells per well) were seeded in 12-well plates (Corning, New York, USA) for 24 h. The LR1+ETEC K88 group was pre-treated by LR1 (1 × 10^9^ cfu/ml) for 4 h firstly, followed by ETEC K88 (1 × 10^8^ cfu/ml) for 4 h. Then, simultaneously, the control group, LR1 group, and ETEC K88 group were treated accordingly for 4 h. To ensure the same CFU of bacteria, we calculated CFU by the dilution coating plate method in each experiment. To knock down the MLCK gene, the IPEC-J2 cells were transfected with MLCK small interfering RNA (siRNA) (50 nM) were performed by Lipofectamine™ 3000 Transfection Reagent (Thermo Fisher Scientific, USA) for 24 h. The si NC sequence is 5’-ACGUGACACGUUCGGAGAATT-3’, and the sequence of siRNA target MLCK 1 gene is 5’-AACCAGGGUGAACACGUCCTT-3’, and that of si MLCK 2 is 5’-UUGGUGCUCACCUUCUUGCTT-3’.

### Cell Viability

The IPEC-J2 cells (1 × 10^4^ cells per well) were seeded in 96-well plates (Corning, New York, USA) and treated with 1 × 10^5^, 1 × 10^6^, 1 × 10^7^, 1 × 10^8^, and 1 × 10^9^ cfu/ml LR1 or ETEC K88. The cell viability was detected by CCK8 assay according to the manufactured protocol (Beyotime, Shanghai, China). Then, IPEC-J2 cells were incubated with 10 μl of CCK8 for 2 h (Beyotime, Shanghai, China). Absorbance at OD_450 nm_ was detected by a fluorescence microplate reader (Bio-Tek, Winooski, USA).

### Intestinal Epithelial Barrier Function

The intestinal epithelial barrier function assay was detected according to our previous reports (21, 22). Briefly, to determine the effect of LR1 and ETEC K88 on the integrity of the IPEC-J2 cell monolayers, the 1 × 10^5^ IPEC-J2 cells/ml were seeded in the Transwell [12-mm-diameter inserts with a 0.4-μm pore size were from Corning, and the transepithelial electrical resistance (TER) was measured by the Millicell-ERS resistance system; Millipore, Bedford, MA]. Additionally, the 500 μg/ml FD4 was added to the upper layer of Transwell for 1.5 h and gathered 100 μl of cell culture medium to detect the FD4 fluorescence using a fluorescence microplate reader (Bio-Tek, Winooski, USA).

### Scanning Electron Microscope (SEM)

We seeded 1 × 10^5^ IPEC-J2 on sterile cover glass in a 12-well cell plate. After being treated with the corresponding treatments, the cells were washed gently with PBS, followed by adding glutaraldehyde in a 12-well cell plate. After fixing for 2 h at room temperature, transfer the petri dish to 4°C. Wash tissue blocks 3 times with 0.1 M PBS for 15 min each. Then, transfer tissue blocks into 1% OsO_4_ in 0.1 M PBS for 1–2 h at room temperature. After that, wash cell blocks 3 times in 0.1 M PBS for 15 min each. Furthermore, the cells were washed with 30% ethanol for 15 min, 50% ethanol for 15 min, 70% ethanol for 15 min, 80% ethanol for 15 min, 90% ethanol for 15 min, and 95% ethanol for 15 min. There were two changes of 100% ethanol for 15 min. Finally, the samples were transferred into isoamyl acetate for 15 min. Specimens are attached to metallic stubs using carbon stickers and sputter-coated with gold for 30 s. The samples were observed and images were taken with the scanning electron microscope.

### Wound Healing Assay

The IPEC-J2 wound healing assay was conducted as previously described (23). Briefly, IPEC-J2 cells were grown in 6-well dishes and the IPEC-J2 monolayer was scratched in a straight line with a p200 pipet tip. The figures were captured with a phase-contrast microscope at the time of scraping and after the corresponding treatment. The percentage of wound closure was calculated using ImageJ. All experiments were repeated 3 times.

### qPCR

Total RNA was extracted from the cell’s samples by Trizol reagent (Invitrogen, Carlsbad, CA). The amount of RNA extracted was determined, and its purity was verified using NanoDrop 1000 (Thermo Fisher Scientific). The cDNA was performed with reagents from EZB Biosystems following conventional protocols. Samples were normalized to GAPDH, and the relative changes of target gene expression were analyzed by the 2^−ΔΔCt^ method. The primers are shown in **Table S1**.

### Western Blot

IPEC-J2 cells were rinsed twice with ice-cold PBS and lysed by RIPA buffer (Invitrogen, USA) containing protease inhibitors, phosphatase inhibitor, and PMSF (Beyotime, Shanghai, China). The supernatant was collected, and a BCA protein assay kit (Beyotime, Shanghai, China) was used to quantify protein concentration. Cell supernatants were resolved on 10% SDS-PAGE gels (Beyotime, Shanghai, China), transferred on PVDF membranes (Millipore), and incubated with the corresponding primary antibodies overnight at 4°C (Claudin-1: ER1906-37, 1:1,000, Huabio; Occludin: ab31721, 1:1,000, Abcam; IL-17A: ER1902-37, 1:1,000, Huabio; beta-actin: 4967, 1:1,000, Cell Signaling). After multiple washing with TBST, the membranes were incubated with secondary antibodies (1:5,000, Huabio, Hangzhou, China) at room temperature for 1 h. Detection was performed by enhanced ECL chemiluminescence (Beyotime, Shanghai, China) and captured by Imaging System. The bands’ intensity was analyzed by ImageJ.

### Data Analysis

Values are given as means ± SEM. Student’s unpaired *t*-test was used to determine statistical significance among each of the two groups. The difference was considered to be significant at *p* < 0.05.

## Results

### Effects of *L. reuteri* LR1 on Cell Viability and Inflammatory Cytokine Expression in IPEC-J2 Cells

**Figure 1:**
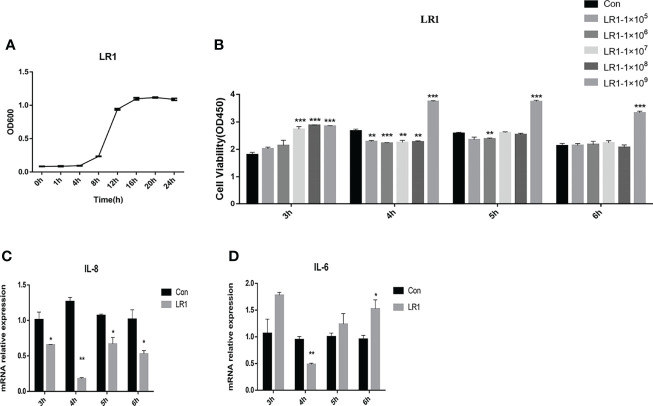
Effects of *L. reuteri* LR1 on cell viability and inflammatory cytokine expression in IPEC-J2 cells. **(A)** The growth curve of *L. reuteri* LR1. **(B)** The effects of different levels of *L. reuteri* LR1 on the viability of IPEC-J2 treated for 3 h, 4 h, 5 h, and 6 h. **(C)** The relative gene expression of IL-8 after being treated with 1 × 10^9^ cfu/ml *L. reuteri* LR1 for 3 h, 4 h, 5 h, and 6 h. **(D)** The relative gene expression of IL-6 after being treated with 1 × 10^9^ cfu/ml *L. reuteri* LR1 for 3 h, 4 h, 5 h, and 6 h. Data presented as mean ± SEM (*n* ≥ 3). ^*^ *p* < 0.05, ^**^ *p* < 0.01, and ^***^ *p* < 0.001 vs. the control group.

The growth curve of LR1 is presented in **Figure 1A**. We optimized the growth condition of LR1 according to the logarithmic phase of LR1. The LR1 strain was cultured overnight at 37°C in MRS for 16 h and collected to treat cells. **Figure 1B** shows that 1 × 10^9^ cfu/ml LR1 treatment for 3 h, 4 h, 5 h, and 6 h significantly enhanced IPEC-J2 cell viability compared with the control group (*p* < 0.05). Furthermore, compared with the control group, the 1 × 10^9^ cfu/ml LR1 treatment for 4 h dramatically decreased the IL-8 and IL-6 expression (**Figures 1C, D**). Thus, in the subsequent experiments, we choose 1 × 10^9^ cfu/ml LR1 pretreatment for 4 h to conduct further experiments.

### Effects of ETEC K88 on Cell Viability and Inflammatory Cytokine Expression in IPEC-J2 Cells

**Figure 2:**
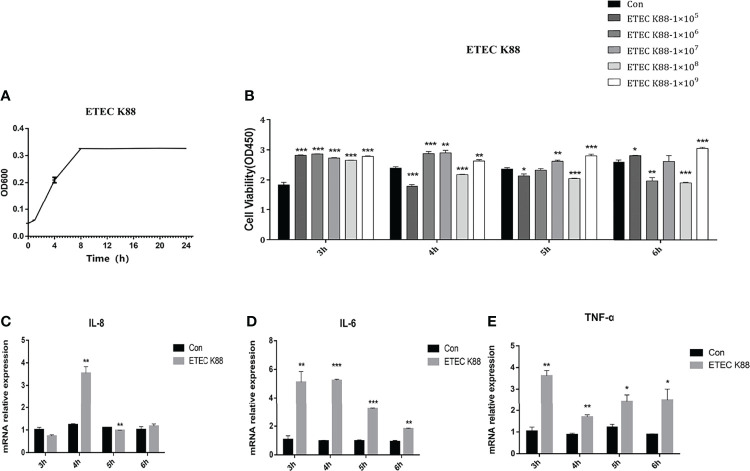
Effects of ETEC K88 on cell viability and inflammatory cytokine expression in IPEC-J2 cells. **(A)** The growth curve of ETEC K88. **(B)** The effects of different levels of ETEC K88 on the viability of IPEC-J2 treated for 3 h, 4 h, 5 h, and 6 h. **(C)** The relative gene expression of IL-8 after being treated with 1 × 10^8^ cfu/ml ETEC K88 for 3 h, 4 h, 5 h, and 6 h. **(D)** The relative gene expression of IL-6 after being treated with 1 × 10^8^ cfu/ml *L. reuteri* LR1 for 3 h, 4 h, 5 h, and 6 h. **(E)** The relative gene expression of TNF-α after being treated with 1 × 10^8^ cfu/ml *L. reuteri* LR1 for 3 h, 4 h, 5 h, and 6 h. Data presented as mean ± SEM (*n* ≥ 3). ^*^ *p* < 0.05,^**^ *p* < 0.01, and ^***^ *p* < 0.001 vs. the control group.

The growth curve of ETEC K88 is presented in **Figure 2A**. We optimized the growth condition of ETEC K88 according to the logarithmic phase of ETEC K88. The ETEC K88 strain was cultured overnight at 37°C in LB for 8 h and collected to treat cells. **Figure 2B** shows that 1 × 10^8^ cfu/ml ETEC K88 treatment for 4 h, 5 h, and 6 h significantly declined the IPEC-J2 cell viability compared to the control group (*p* < 0.05). Additionally, compared with the control group, the 1 × 10^8^ cfu/ml ETEC K88 treatment for 4 h dramatically enhanced the IL-8, IL-6, and TNF-α expression (*p* < 0.05) (**Figures 2C, D**). Thus, in the subsequent experiments, we choose 1 × 10^8^ cfu/ml ETEC K88 treatment for 4 h to perform further experiments.

### Effects of *L. reuteri* LR1 on Cell Adherence and Wound Healing in IPEC-J2 Cells Treated With ETEC K88

**Figure 3:**
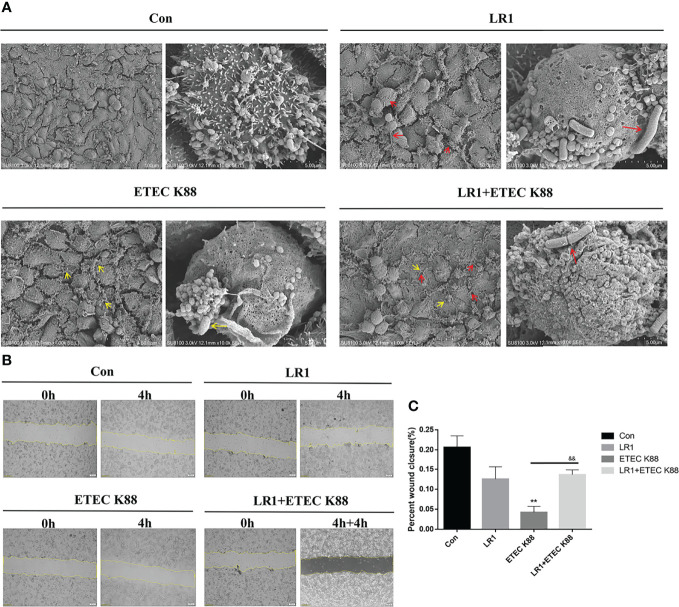
Effects of *L. reuteri* LR1 on cell adherence and wound healing in IPEC-J2 cells treated with ETEC K88. **(A)** The scanning electron microscope (SEM) of IEPC-J2 cells pretreated with *L. reuteri* LR1 (1 × 10^9^ cfu/ml) for 4 h followed by ETEC K88 (1 × 10^8^ cfu/ml) challenged for 4 h. **(B)** *L. reuteri* LR1 promotes cell motility as shown by *in vitro* scratch assays in IPEC-J2 cells challenged with ETEC K88. Live-cell images were taken right after the “scratches” were created (0 h) and at 8 h post-treatment (or not) of *L. reuteri* LR1 (1 × 10^9^ cfu/ml) and ETEC K88 (1 × 10^8^ cfu/ml). **(C)** Quantification of percent wound closure was performed by detecting the decline in the denuded area at 8 h. Cells were treated with *L. reuteri* LR1 (1 × 10^9^ cfu/ml) for 4 h, and then followed by ETEC K88 (1 × 10^8^ cfu/ml) for 4 h. Data presented as mean ± SEM (*n* = 3). ^**^ *p* < 0.01 vs. the control group, ^&&^ *p* < 0.01 vs. the ETEC K88 group. Red arrows in “LR1 and LR1+ETEC K88” represent *Lactobacillus reuteri 1* (LR1), and yellow arrows in “*ETEC K88 and LR1+ETEC K88*” represent *E. coli* (ETEC K88).

We utilized the SEM to detect the adherence of LR1 and ETEC K88 on IPEC-J2. We found that pretreatment with LR1 inhibited the ETEC K88 that adhered on IPEC-J2 (**Figure 3A**). Furthermore, compared with the control group, the scratch edge of IPEC J2 cells retreated after ETEC K88 treatment. Compared with the ETEC K88 group, pretreatment with LR1 alleviated the scratch injury of IPEC J2 cells (*p* < 0.05) (**Figure 3B**).

### Effects of *L. reuteri* LR1 on Intestinal Epithelial Barrier Function and Tight Junction Protein Expression in IPEC-J2 Cells Treated With ETEC K88

**Figure 4:**
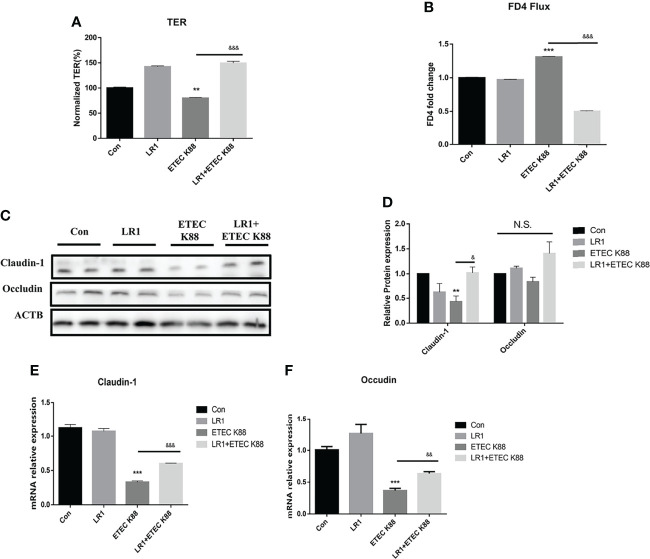
Effects of *L. reuteri* LR1 on intestinal epithelial barrier function and tight junction protein expression in IPEC-J2 cells treated with ETEC K88. **(A)** The effect of *L. reuteri* LR1 (1 × 10^9^ cfu/ml) on transepithelial electrical resistance (TER) in IPEC-J2 cells treated with ETEC K88 (1 × 10^8^ cfu/ml). **(B)** The effect of *L. reuteri* LR1(1 × 10^9^ cfu/ml) on FD4 flux in IPEC-J2 cells treated with ETEC K88 (1 × 10^8^ cfu/ml). **(C)** The effect of *L. reuteri* LR1 (1 × 10^9^ cfu/ml) on tight junction protein expression in IPEC-J2 cells treated with ETEC K88 (1 × 10^8^ cfu/ml). **(D)** Quantification of tight junction protein expression. **(E)** The effect of *L. reuteri* LR1 (1 × 10^9^ cfu/ml) on Claudin-1 gene expression in IPEC-J2 cells treated with ETEC K88 (1 × 10^8^ cfu/ml). **(F)** The effect of *L. reuteri* LR1 (1 × 10^9^ cfu/ml) on Occludin gene expression in IPEC-J2 cells treated with ETEC K88 (1 × 10^8^ cfu/ml). Cells were treated with *L. reuteri* LR1 (1 × 10^9^ cfu/ml) for 4 h, and then followed by ETEC K88 (1 × 10^8^ cfu/ml) for 4 h. Data presented as mean ± SEM (*n* = 3). ^**^ *p* < 0.01 and ^***^ *p* < 0.001 vs. the control group, ^&&^ *p* < 0.01 and ^&&&^ *p* < 0.001 vs. the ETEC K88 group. N.S., not significant.

We measured the TER, FD4 flux, and tight junction protein expression by Western blotting to investigate the effects of LR1 on intestinal epithelial barrier function in IPEC-2 cells. As shown in **Figure 4A**, compared with the control group, ETEC K88 treatment significantly reduced the TER of IPEC J2 cells, and LR1 pretreatment significantly increased the TER after ETEC K88 treatment (*p* < 0.05), while, as presented in **Figure 4B**, in comparison with the control group, ETEC K88 treatment significantly enhanced the FD4 flux of IPEC J2 cells, and LR1 pretreatment significantly declined the FD4 flux after ETEC K88 treatment (*p* < 0.05). Furthermore, ETEC K88 treatment significantly declined the Claudin-1 expression compared to the control group. Additionally, LR1 pretreatment dramatically enhanced the Claudin-1 expression compared with the ETEC K88 group (*p* < 0.05) (**Figures 4C, D**). Consistently, LR1 pretreatment reversed the declined mRNA levels of Occludin and Claudin-1 induced by ETEC K88 challenge (*p* < 0.05) (**Figures 4C, D**).

### Effects of *L. reuteri* LR1 on Inflammatory Cytokine Expression and MLCK Signal Pathway Expression in IPEC-J2 Cells Treated With ETEC K88

**Figure 5:**
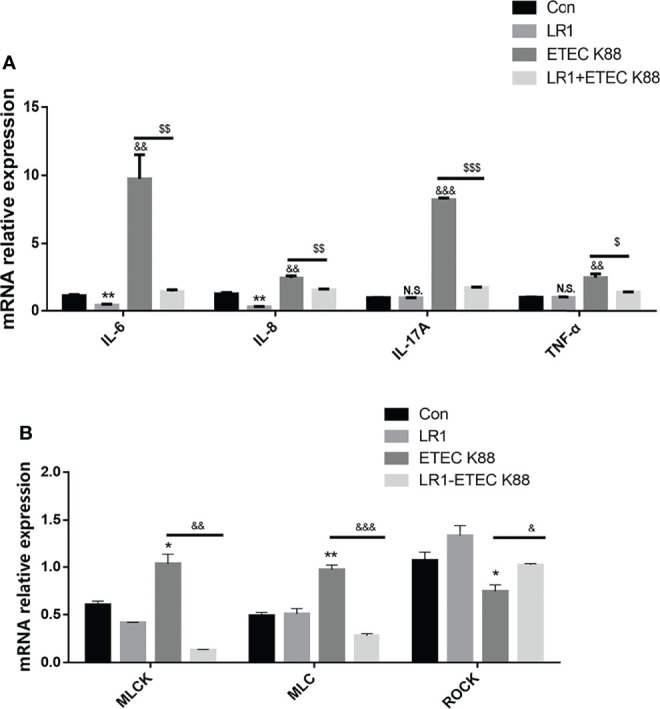
Effects of *L. reuteri* LR1 on inflammatory cytokine expression and MLCK signal pathway expression in IPEC-J2 cells treated with ETEC K88. **(A)** The effects of *L. reuteri* LR1 (1 × 10^9^ cfu/ml) on IL-8, IL-17A, IL-6, and TNF-α gene expression in IPEC-J2 cells treated with ETEC K88 (1 × 10^8^ cfu/ml). Cells were treated with *L. reuteri* LR1 (1 × 10^9^ cfu/ml) for 4 h, and then followed by ETEC K88 (1 × 10^8^ cfu/ml) for 4 h. Data presented as mean ± SEM (*n* = 3). **(B)** The effects of *L. reuteri* LR1 (1 × 10^9^ cfu/ml) on MLCK, MLC, and ROCK gene expression in IPEC-J2 cells treated with ETEC K88 (1 × 10^8^ cfu/ml). Cells were treated with *L. reuteri* LR1 (1 × 10^9^ cfu/ml) for 4 h, and then followed by ETEC K88 (1 × 10^8^ cfu/ml) for 4 h. Data presented as mean ± SEM (*n* = 3). ^*^ *p* < 0.05, ^**^ *p* < 0.01, and ^***^ *p* < 0.001 vs. the control group; ^&^ *p* < 0.05, ^&&^ *p* < 0.01, and ^&&&^ *p* < 0.001 vs. the control group; ^$^ *p* < 0.05, ^$$^ *p* < 0.01, and ^$$$^ *p* < 0.001 vs. the ETEC K88 group. N.S., not significant.

We recollected the samples and measured the IL-6, IL-8, and TNF-α level to investigate the effects of LR1 on inflammatory cytokine expression. As shown in **Figure 5A**, compared with the control group, IL-6 and IL-8 levels were declined in IPEC-J2 after LR1 treatment, while IL-17A and TNF-α levels were not influenced by LR1 treatment in IPEC-J2 (*p* < 0.05) (**Figure 5A**). Furthermore, in comparison with the control group, ETEC K88 treatment significantly enhanced the IL-6, IL-8, IL-17A, and TNF-α expression in IPEC-J2 (*p* < 0.05) (**Figure 5A**). Meanwhile, compared with ETEC K88 group, LR1 pretreatment dramatically declined IL-6, IL-8, and TNF-α levels in IPEC-J2 treated with ETEC K88 (*p* < 0.05) (**Figure 5A**). Consistently, previous studies reported that LR alleviated inflammation by reducing the production of pro-inflammatory cytokines. Moreover, ETEC K88-treated IPEC-J2 cells had a higher MLCK level, higher MLC levels, and lower ROCK levels than the control group (*p* < 0.05) (**Figure 5B**), while LR1 pretreatment significantly declined MLCK and MLC expression and enhanced ROCK level, in comparison with the ETEC K88 group (*p* < 0.05) (**Figure 5B**).

### Knockdown of MLCK Promotes the Effects of *L. reuteri* LR1 on the Inflammatory Response and Barrier Function in IPEC-J2 Cells Treated With ETEC K88

**Figure 6:**
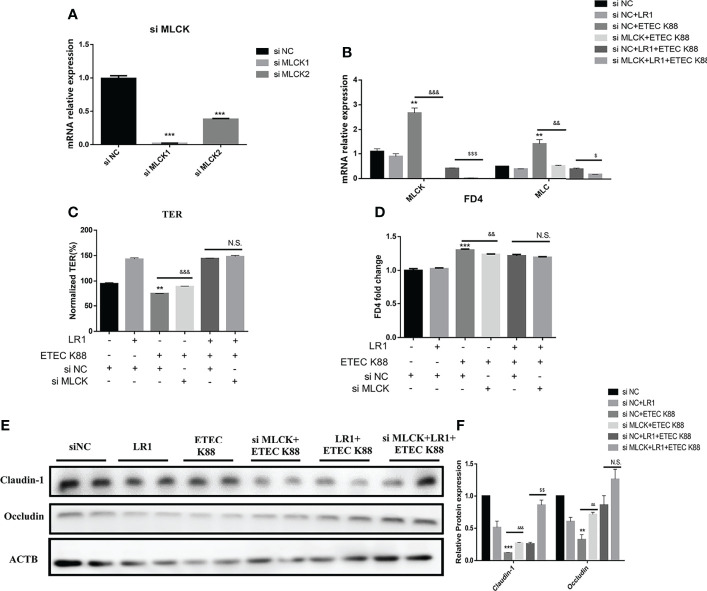
Knockdown MLCK promotes the effects of *L. reuteri* LR1 on the inflammatory response and barrier function in IPEC-J2 cells treated with ETEC K88. **(A)** Relative expression of MLCK in IPEC-J2 after being treated with si Negative Control (si NC), si MLCK 1, and si MLCK 2. **(B)** Gene expression of MLCK and MLC determined by qPCR in IPEC-J2 cells transfected with negative control siRNA or siRNA targeting MLCK. **(C)** TER of IPEC-J2 cells transfected with negative control siRNA or siRNA targeting MLCK. **(D)** FD4 flux of IPEC-J2 cells transfected with negative control siRNA or siRNA targeting MLCK. **(E)** Protein expression of Occludin and Claudin-1 determined by Western blotting in IPEC-J2 cells transfected with control siRNA or siRNA targeting MLCK. **(F)** Quantification of protein expression of Occludin and Claudin-1. Cells were treated with *L. reuteri* LR1 (1 × 10^9^ cfu/ml) for 4 h, and then followed by ETEC K88 (1 × 10^8^ cfu/ml) for 4 h. siRNA transfection for 24 h before all treatments. Data presented as mean ± SEM (*n* = 3). ^**^ *p* < 0.01 and ^***^ *p* < 0.001 vs. the control group, ^&&^ *p* < 0.01 and ^&&&^ *p* < 0.001 vs. the ETEC K88 group, ^$$^ *p* < 0.01 vs. the siNC+LR1+ETEC K88 group. N.S., not significant.

To elucidate whether LR1 plays a protective role through the regulation of the MLCK signal pathway, we used the siRNA to knock down the MLCK gene in IPEC-J2 cells (**Figure 6A**). Interestingly, we found that depletion of MLCK significantly declined MLC expression in IPEC-J2 challenged with *ETEC K88* than the si NC+ETEC K88 group (*p* < 0.05) (**Figure 6B**). Furthermore, in comparison with the si NC+LR1+ETEC K88 group, depletion of MLCK significantly declined MLCK and MLC expression (*p* < 0.05) (**Figure 6B**). The TER of IPEC-J2 in the si MLCK+ETEC K88 group was higher and the FD4 flux of IPEC-J2 in the si MLCK+ETEC K88 group was lower compared with the si NC+ETEC K88 group (*p* < 0.05) (**Figures 6C, D**), while there is no significant difference in TER and FD4 permeability between the si NC+LR1+ETEC K88 group and the si MLCK+LR1+ETEC K88 group (*P* > 0.05) (**Figures 6C, D**). Compared with the si NC+ETEC K88 group, the si MLCK+ETEC group had a higher expression of Claudin-1 and Occludin (*p* < 0.05) (**Figures 6E, F**). These data revealed that knockdown of the MLCK inhibited the intestinal barrier dysfunction induced by ETEC K88 challenge. Consistently, depletion of MLCK enhanced Claudin-1 level in comparison to the si NC+LR1+ETEC K88 group (*p* < 0.05) (**Figures 6E, F**). We added the inflammatory cytokine expression data in MLCK Knockdown IPEC-J2 (**Supplementary Figure S3**). We found that depletion of MLCK significantly declined IL-8 and TNF-α expression in IPEC-J2 challenged with *ETEC K88* compared to the si NC+ETEC K88 group (*p* < 0.05) (**Supplementary Figure S3**). Furthermore, in comparison with the si NC+LR1+ETEC K88 group, depletion of MLCK significantly declined IL-8 and IL-6 expression (*p* < 0.05), suggesting that the MLCK signal pathway is involved in immune pathogenesis after being treated with ETEC K88 (**Supplementary Figure S3**).

## Discussion

Intestinal epithelial barrier injury disrupts immune homeostasis and leads to many intestinal disorders (24–26). *L. reuteri* strains are important members of the commensal microbiota in the intestines of piglets, which can influence the immune system development and intestinal barrier (7, 8). However, the effects of *L. reuteri* LR1 on intestinal integrity and inflammatory response and the underlying mechanisms are yet unknown. Thus, in this study, we determined the effects of LR1 on the ETEC K88-induced intestinal epithelial injury on the inflammatory response, intestinal epithelial barrier function, and the MLCK signal pathway and its underlying mechanism.

Several lines of evidence revealed that *L. reuteri* has the potential to reduce mucosal leakage and protect the cell against death induced by stressors (27, 28). Xie et al. reported that *L. reuteri* stimulates intestinal epithelial proliferation and induces differentiation into goblet cells in young chickens (27). Wu et al. found that *L. reuteri* helps to maintain intestinal epithelium renewal and homeostasis and heals pathological damage (28). Our results showed that 1 × 10^9^ cfu/ml LR1 treatment significantly enhanced IPEC-J2 cell viability compared with the control group. Cytokines, which modulate intestinal mucosal inflammation and epithelial integrity, are known to be involved in the pathology development of inflammatory bowel disease (29–31). Wang et al. reported that *L. reuteri* can ameliorate intestinal inflammation in dextran sulfate sodium-induced colitis in mice, indicated by decreased levels of the proinflammatory cytokines IFN-γ, TNF-α, IL-1β, IL-6, and IL-17A in the colon tissue and serum of mice (32). The present study demonstrated that the 1 × 10^9^ cfu/ml LR1 treatment for 4 h dramatically decreased the IL-8 and IL-6 expression.

Many studies reported that *L. reuteri* had a strong pathogen inhibition effect through microbe interactions, inhibiting the adhesion of pathogenic bacteria, competition for nutrients, and binding sites (33, 34). Zhang et al. reported that *L. reuteri* adhered to IPEC-J2 and Caco-2 cells *via* glyceraldehyde-3-phosphate dehydrogenase (35). Walsham et al. indicated that *L. reuteri* can bind to the intestinal layer that led to declined enteropathogenic *E. coli* adherence to small intestinal biopsy epithelium (34). Consistently, we utilized the SEM to detect the adherence of LR1 and ETEC K88 on IPEC-J2, and we found that pretreatment with LR1 inhibited the ETEC K88 that adhered on IPEC-J2. Some studies reported that *L. reuteri* could promote cell renewal and wound healing (36, 37). In this study, the function of IPEC-J2 cell migration and the ability of wound healing were estimated by the scratch-simulated wound migration assay. Our data indicated that compared with the ETEC K88 group, pretreatment with LR1 alleviated the scratch injury of IPEC J2 cells. These data suggested that LR1 pretreatment could inhibit ETEC K88 adhesion and promote cell migration in the ETEC K88-challenged model.

The gut epithelium serves as the most important barrier to prevent endogenous and exogenous harmful antigens and pathogens, and the tight junction is a basis for intestinal barrier function (26, 38). In this study, the tight junction of intestinal epithelial cells and the paracellular permeability of the gut were indirectly reflected by the TER and FD4 flux (21, 22). The data indicated that LR1 pretreatment significantly reversed the declined TER and tight junction protein level and enhanced the induction by ETEC K88 treatment. Our previous study reported that LR1 supplementation at 5 × 10^10^ cfu/kg could improve the growth performance, intestinal morphology, and intestinal barrier function in weaned pigs (17). Similarly, Li et al. indicated that *L. reuteri* improves gut barrier function and affects the diurnal variation of the gut microbiota in mice fed a high-fat diet (39). Cui et al. reported that *L. reuteri* ZJ617 maintains intestinal integrity *via* regulating tight junction, autophagy, and apoptosis in mice challenged with lipopolysaccharide (40). Several studies reported that there is a strong connection between the enhanced proinflammatory cytokine level and gut integrity damage (25, 41). Several lines of evidence revealed that the inflammatory response of intestinal epithelial cells is closely related to the expression of inflammatory cytokines (42, 43). Our data found that LR1 pretreatment dramatically declined the proinflammatory cytokines IL-8, IL-17A, IL-6, and TNF-α levels compared with the ETEC K88 group. Consistently, previous studies reported that LR alleviated inflammation by reducing the production of pro-inflammatory cytokines. Tang et al. found that LR treatment significantly decreased IL-1b, IL-6, TNF-α, and IFN-γ expression in the jejunum of weaning piglets (44). Hsieh et al. reported that *L. reuteri* GMNL-263 decreased serum TNF-α and IL-6 levels in mice fed with a high-fat diet (45). Karimi et al. pointed out that pretreatment with *L. reuteri* decreased IL-6 and TNF-α levels, and enhanced a longer isoform of ZO-1 and maintained E-cadherin expression. The interleukin-17 (IL-17) family consists of a subset of cytokines that participate in both acute and chronic intestinal inflammatory responses (46). Though Th17 cells were thought as a major source of IL-17A, IL-17A can also be produced by other cell types (47). We detected the IL-17A protein levels in IPEC-J2 and used the mouse colonic tissue protein results as a positive control. As shown in **Supplementary Figure S2A**, we confirmed that IL-17A can be expressed in IPEC J2 cells (**Supplementary Figure S2A**). Consistently, Zhang et al. found that IL-17 can express in IPEC-J2 cells (48). Furthermore, Yu et al. found that TLR5-mediated IL-17C expression in IPEC-J2 enhances immune responses in the intestinal epithelium against ETEC infection (49). These data revealed that LR1 pretreatment could reverse the disrupted intestinal barrier function and inflammatory response induced by ETEC K88 challenge.

The MLCK signal pathway has been widely reported to play a critical function in regulating the tight junction proteins’ reorganization, *via* promoting the enhanced contraction and tonicity of actomyosin before tight junction (50, 51). Then, the activated MLCK signal pathway led to the tight junction disrupted arrangement and enhanced the intestinal paracellular permeability (20, 52, 53). Moreover, the MLCK signal pathway is involved in mediating pro-inflammatory cytokine expressions, such as IL-β, IL-6, and IL-8 (54, 55). Several studies reported that there is a strong connection between the enhanced proinflammatory cytokines’ level and the immune pathogenesis of intestinal inflammatory (29). Several lines of evidence revealed that *E. coli* is an important pathogenic bacteria to induce intestinal inflammatory diseases, indicated by the enhanced proinflammatory cytokine expression and cell damage (56). Su et al. found that targeted inhibition of intestinal epithelial MLCK may be therapeutically effective in immune-mediated inflammatory bowel disease, particularly in preventing reactivation of quiescent inflammatory bowel disease (57). Our data suggested that LR1 pretreatment reversed the enhanced MLCK and MLC expression and decreased ROCK level induced by ETEC K88-challenged IPEC-J2 cells. In addition, we also found that depletion of MLCK significantly enhanced Claudin-1 level and declined IL-8 and TNF-α level in IPEC-J2 pretreated with LR1 followed by challenging with ETEC K88. These results suggest that the inhibited MLCK signal pathway may be responsible for *L. reuteri* intestinal protection functions and alleviating inflammatory response in IPEC-J2 cells.

In conclusion, our work proposes that *L. reuteri* LR1 can improve intestinal epithelial barrier function and declined inflammatory response through suppressing the MLCK signal pathway. These results provide us with new insights into the protective effect of *L. reuteri* on the gut epithelial integrity and inflammation response, giving us a potential therapeutic function for the therapy of intestinal inflammatory diseases.

## Data Availability Statement

The datasets presented in this study can be found in online repositories. The names of the repository/repositories and accession number(s) can be found below: Scan electron microscope (SEM) data has been uploaded to FigShare. The DOI number is https://doi.org/10.6084/m9.figshare.19880665.v1.

## Author Contributions

Conceptualization: ZJ, SC, and LW. Methodology: JG and SC. Software: JG. Validation: HX, SH, KY, and KH. Formal analysis, SC and JG. Data curation: JG. Writing—original draft preparation: SC and JG. Writing—review and editing: LW. Visualization: SC. Supervision: ZJ and LW. Funding acquisition: ZJ, LW, and SC. All authors have reviewed and approved the final manuscript.

## Funding

This study was supported by the National Natural Science Foundation of China (32172777), the China Postdoctoral Science Foundation (2021M700894), the Guangdong Basic and Applied Basic Research Foundation (2022A1515011406, 2021A1515110636), and the Science and Technology Program of Guangdong Academy of Agricultural Sciences (R2020PY-JG009, 202106TD).

## Conflict of Interest

The authors declare that the research was conducted in the absence of any commercial or financial relationships that could be construed as a potential conflict of interest.

## Publisher’s Note

All claims expressed in this article are solely those of the authors and do not necessarily represent those of their affiliated organizations, or those of the publisher, the editors and the reviewers. Any product that may be evaluated in this article, or claim that may be made by its manufacturer, is not guaranteed or endorsed by the publisher.

## Supplementary Material

Supplementary Figure S1 Diagram for cell treatments. The IPEC-J2 cells (1×105 cells per well) were seeded in 12-well plates (Corning, New York, USA) for 24h. And the LR1 + ETEC K88 group was pre-treated by LR1 (1 × 109 cfu/mL) for 4h firstly, followed by ETEC K88 (1 × 108 cfu/mL) for 4h. And then, simultaneously, the control group, LR1 group and ETEC K88 group were treated accordingly for 4h. Four independent experiments were conducted to verify the results and three replicates per group in every independent test.

Supplementary Figure S2 IL-17A protein expression. (A) IL-17A proteins expression in mouse colon and IPEC-J2 cells.

Supplementary Figure S3 Knockdown MLCK inhibition the effects of L. reuteri LR1 on the inflammatory response in IPEC-J2 Cells treated with ETEC K88. (A) Gene expression of IL-8 determined by qPCR in IPEC-J2 cells transfected with negative control siRNA or siRNA targeting MLCK. (B) Gene expression of TNF-α determined by qPCR in IPEC-J2 cells transfected with negative control siRNA or siRNA targeting MLCK. Data presented as mean ± SEM (n=3). ***p < 0.001 vs. Control group, ##p < 0.01 and ###p < 0.001 vs. ETEC K88 group, &p < 0.05 and &&p < 0.01 vs. siNC+LR1+ETEC K88 group.
