# Supplementary figures and images for: Lactobacillus reuteri 1 Enhances Intestinal Epithelial Barrier Function and Alleviates the Inflammatory Response Induced by Enterotoxigenic Escherichia coli K88 via Suppressing the MLCK Signaling Pathway in IPEC-J2 Cells
Source: Front Immunol. 2022 Jul 14;13:897395. doi: 10.3389/fimmu.2022.897395 (PMC9331657; doi:10.3389/fimmu.2022.897395)

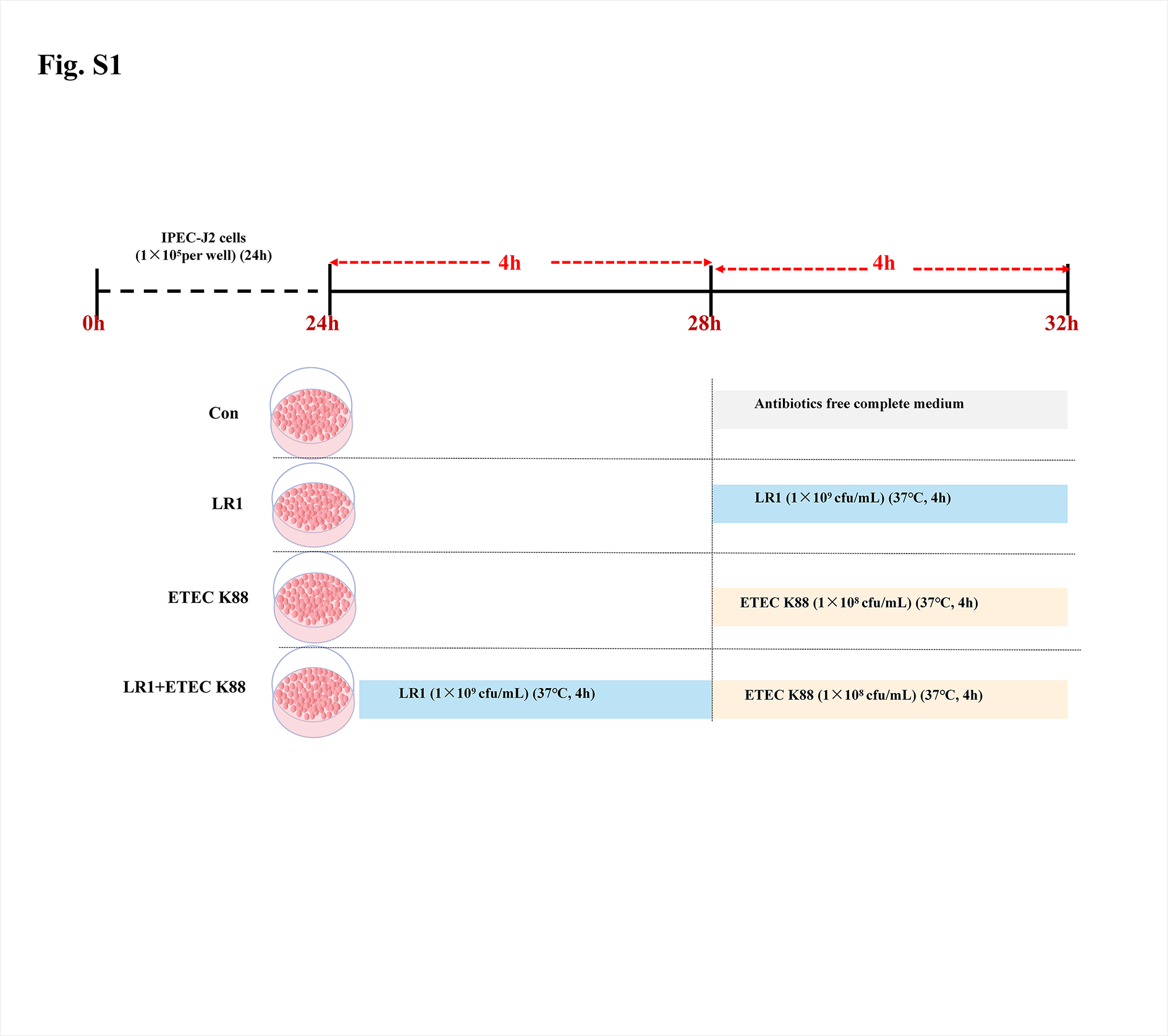

Supplement: Supplementary Figure S1 — Diagram for cell treatments. The IPEC-J2 cells (1×10^5^ cells per well) were seeded in 12-well plates (Corning, New York, USA) for 24h. And the LR1 + ETEC K88 group was pre-treated by LR1 (1 × 10^9^ cfu/mL) for 4h firstly, followed by ETEC K88 (1 × 10^8^ cfu/mL) for 4h. And then, simultaneously, the control group, LR1 group and ETEC K88 group were treated accordingly for 4h. Four independent experiments were conducted to verify the results and three replicates per group in every independent test. [file Image_1.tif]

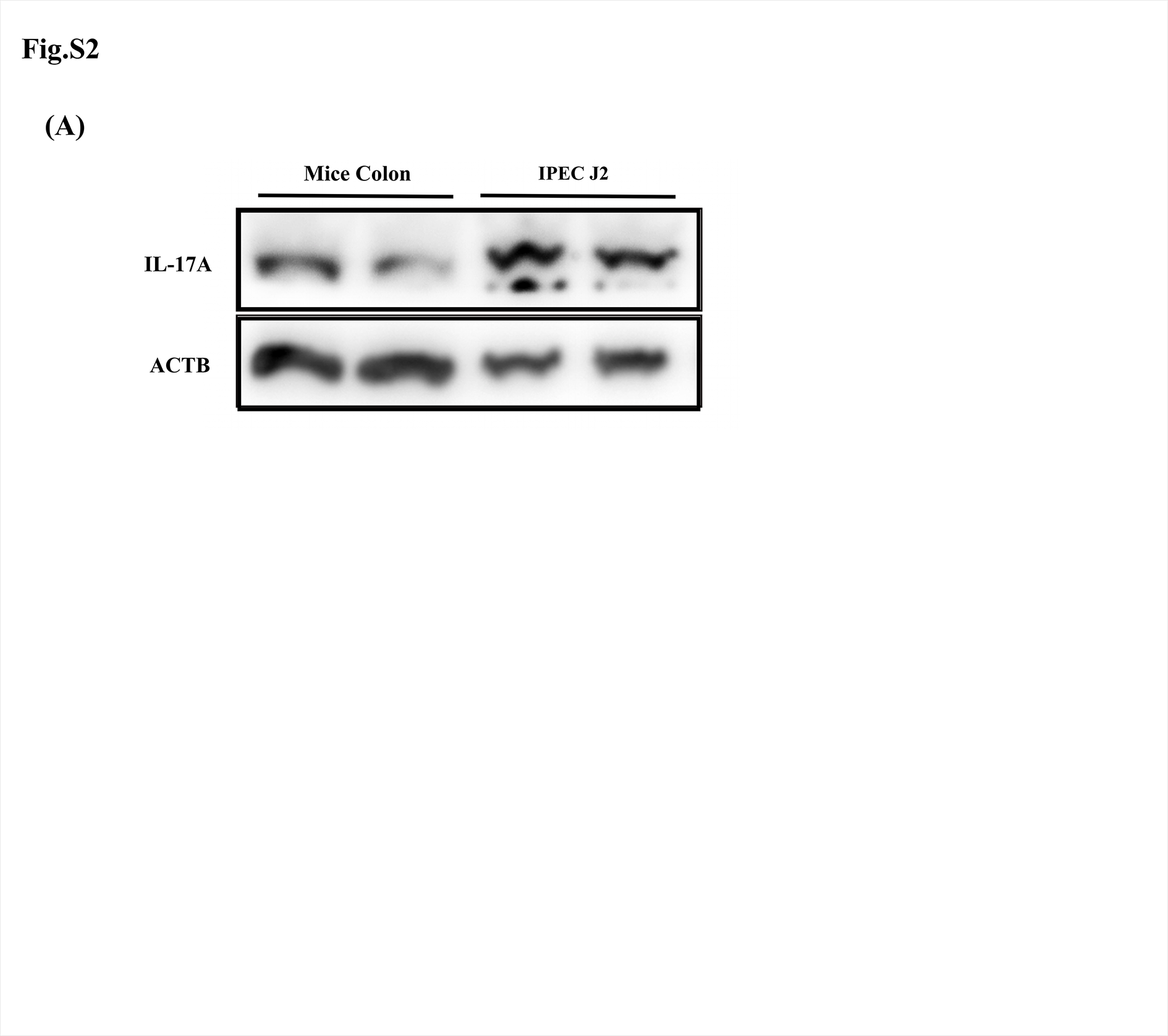

Supplement: Supplementary Figure S2 — IL-17A protein expression. (A) IL-17A proteins expression in mouse colon and IPEC-J2 cells. [file Image_2.tif]

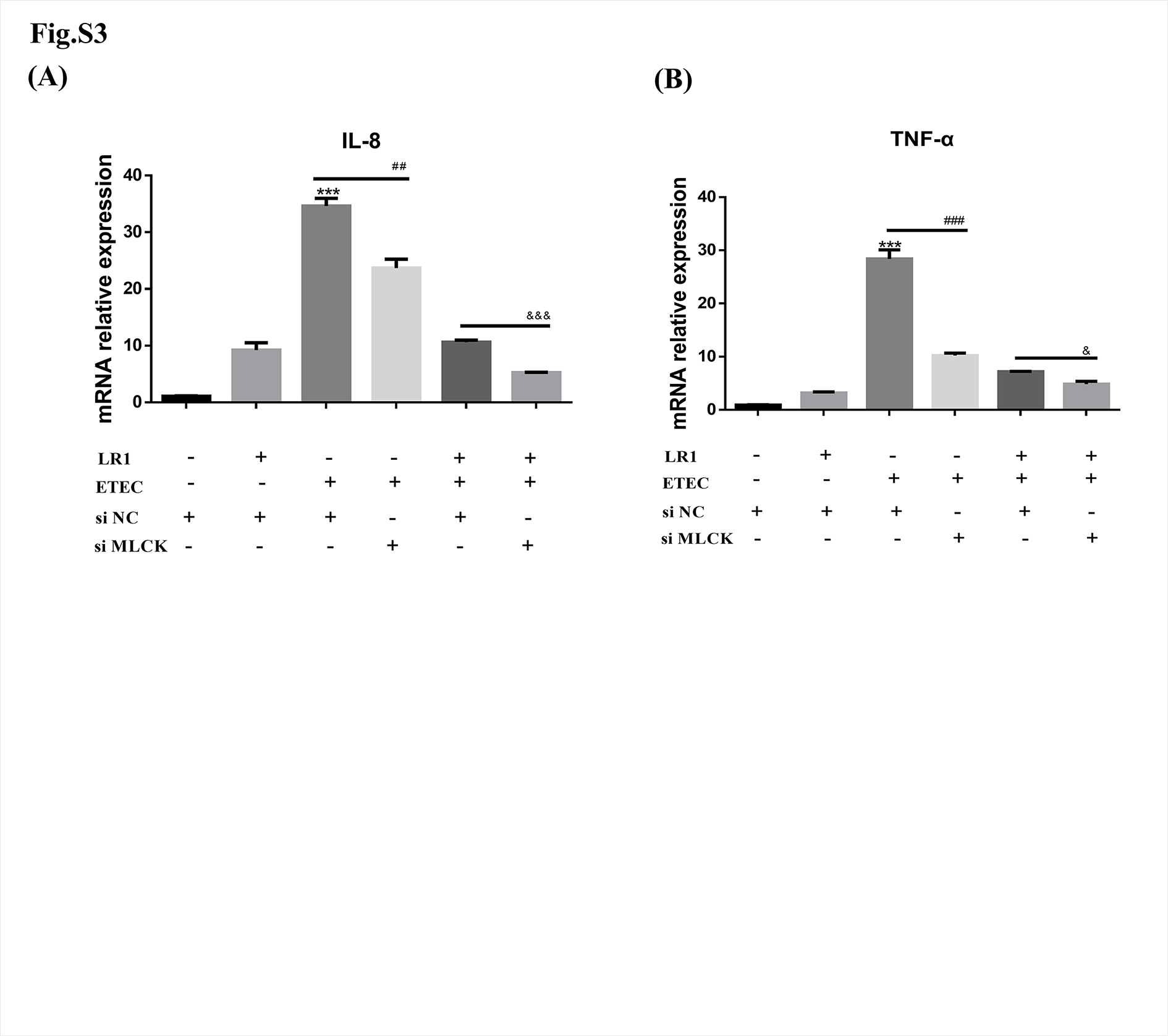

Supplement: Supplementary Figure S3 — Knockdown MLCK inhibition the effects of L. reuteri LR1 on the inflammatory response in IPEC-J2 Cells treated with ETEC K88. (A) Gene expression of IL-8 determined by qPCR in IPEC-J2 cells transfected with negative control siRNA or siRNA targeting MLCK. (B) Gene expression of TNF-α determined by qPCR in IPEC-J2 cells transfected with negative control siRNA or siRNA targeting MLCK. Data presented as mean ± SEM (n=3). ***p < 0.001 vs. Control group, ##p < 0.01 and ###p < 0.001 vs. ETEC K88 group, &p < 0.05 and &&p < 0.01 vs. siNC+LR1+ETEC K88 group. [file Image_3.tif]
