# Supplementary material for: Lactobacillus reuteri 1 Enhances Intestinal Epithelial Barrier Function and Alleviates the Inflammatory Response Induced by Enterotoxigenic Escherichia coli K88 via Suppressing the MLCK Signaling Pathway in IPEC-J2 Cells
Source: Front Immunol. 2022 Jul 14;13:897395. doi: 10.3389/fimmu.2022.897395 (PMC9331657; doi:10.3389/fimmu.2022.897395)
Supplement: Supplementary file 4 [file Table_1.docx]

**Table s1.Primer sequences used for real-time PCR.**

| Gene | 5’-Primer (F) | 3’-Primer (R) | Accession number | Length |
| --- | --- | --- | --- | --- |
| GAPDH | CATCCTGGGCTACACTGAGG | TGGTCGTTGAGGGCAATG | XM_021091114.1 | 102 |
| IL-6 | TGGCTACTGCCTTCCCTACC | CAGAGATTTTGCCGAGGATG | NM_001252429.1 | 132 |
| IL-8 | TTCGATGCCAGTGCATAAATA | CTGTACAACCTTCTGCACCCA | NM_213467.1 | 176 |
| IL-17a | ACTCCAAACGCTTCACCTCA | TCAGCATTGATACAGCCCGA | NM_001005729.1 | 112 |
| TNF-α | AACCTCCTCTCTGCCATCAA | TGCCCAGATTCAGCAAAGTC | NM_214022.1 | 173 |
| ZO-1 | AGCCCGAGGCGTGTTT | GGTGGGAGGATGCTGTTG | XM_021098896.1 | 147 |
| Claudin-1 | GACTCCTTGCTGAATCTGA | GCACCTCATCATCTTCCAT | XM_005666863.2 | 139 |
| Occludin | GCACCCAGCAACGACAT | CATAGACAGAATCCGAATCAC | XM_005672525.3 | 144 |
| MLCK | CTCCAAGGACCGGATGAA | CCACTGAGCCCTGAGATCAT | XM_001929078.6 | 114 |
| ROCK | TCAGCAGTGACATAGACAG | CCAGGCGAGTATTAACAGA | XM_021087837.1 | 309 |
